# Breakdown in informational continuity of care during hospitalization of older home-living patients: a case study

**DOI:** 10.5334/ijic.1525

**Published:** 2014-05-12

**Authors:** Rose Mari Olsen, Ove Hellzén, Liv Heidi Skotnes, Ingela Enmarker

**Affiliations:** Faculty of Health and Science, Nord-Trøndelag University College, Namsos, Norway; Centre for Care Research Mid-Norway, Steinkjer, Norway; Department of Health Sciences, Mid-Sweden University, Sundsvall, Sweden; Department of Medicine, Division of Geriatrics, Nord-Trøndelag HealthTrust, Namsos, Norway; Faculty of Health and Science, Nord-Trøndelag University College, Namsos, Norway; Centre for Care Research Mid-Norway, Steinkjer, Norway

**Keywords:** older people, transfer, nursing information exchange, hospitalization, home care

## Abstract

**Introduction:**

The successful transfer of an older patient between health care organizations requires open communication between them that details relevant and necessary information about the patient's health status and individual needs. The objective of this study was to identify and describe the process and content of the patient information exchange between nurses in home care and hospital during hospitalization of older home-living patients.

**Methods:**

A multiple case study design was used. Using observations, qualitative interviews and document reviews, the total patient information exchange during each patient's episode of hospitalization (*n* = 9), from day of admission to return home, was captured.

**Results:**

Information exchange mainly occurred at discharge, including a discharge note sent from hospital to home care, and telephone reports from hospital nurse to home care nurse, and meetings between hospital nurse and patient coordinator from the municipal purchaser unit. No information was provided from the home care nurses to the hospital nurses at admission. Incompleteness in the content of both written and verbal information was found. Information regarding physical care was more frequently reported than other caring dimensions. Descriptions of the patients’ subjective experiences were almost absent and occurred only in the verbal communication.

**Conclusions:**

The gap in the information flow, as well as incompleteness in the content of written and verbal information exchanged, constitutes a challenge to the continuity of care for hospitalized home-living patients. In order to ensure appropriate nursing follow-up care, we emphasize the need for nurses to improve the information flow, as well as to use a more comprehensive approach to older patients, and that this must be reflected in the verbal and written information exchange.

## Introduction

The importance of continuity of care for older patients has long been recognized. Because of the complexity of their health conditions [1, 2], older patients tend to have more frequent hospital admissions [3], use a higher number of other health care services [4], and thus experience a great array of care pathways across health care levels. For older patients, continuity of care is particularly important during care transfer [5–7]. Yet it is becoming increasingly apparent that their health care providers struggle to coordinate health information exchange across care settings and are more likely to have inaccurate and incomplete clinical information [8–14]. Nurses play an important role in obtaining and sharing relevant and necessary information among providers to ensure continuity of care. In this study, we report findings from a study dealing with nurses’ information exchange during older patients’ transfer.

Continuity of care has been a subject for research in many years, and as a concept, it has been defined in a variety of ways [15]. We apply the definition given by Haggerty et al. [16], which takes into account the subjective point of view of patients and providers: ‘Continuity is the degree to which a series of discrete health care events is experienced as coherent and connected and consistent with the patient's medical needs and personal context’. Three essential types of continuity are described, including: relational, management and informational [16, 17]. While the relational type concerns the patient's ongoing engagement with one or more providers, the management and informal types are related to the coordination aspect of continuity. The latter type will be our focus for the remainder of this study. Informational continuity of care refers to how well a patient's health information is able to ‘follow him/her’ throughout the pathway of care, including over time and between providers in different settings [16]. Given this definition and our focus here on nurses’ information exchange, for the purpose of this study we consider ‘information’ to be facts about nursing care and patient's status that may be conveyed verbally, or in written (including electronically) form between home care nurses and hospital nurses.

In order to ensure continuity of care, nurses in Norway are obligated by law to document care provided in the patient record, as well as exchange relevant and necessary information [18]. This obviously also includes passing information across health care settings during patient transfer. The requirements for in what way the information should be exchanged differ, however, depending on what kind of health care setting the patient is transferred from. While transfer from institutions, such as hospitals and nursing homes, requires a written (or electronically) summary of the patient record [19], the act does not have any requirements for in what form the information should be provided by the home health care. Recently, we reported a study including older patients transferred from home care to hospital and back to home care again after hospitalization [20]. We found that there was only one instance out of 102 patients in which a nursing transfer document was exchanged at hospital admission, while discharge notes were present in 69% (*n* = 70). A lack of nursing transfer documents sent between hospital and primary care has been reported also by other researchers [13, 21]. Simultaneously, it has been reported that information exchange between nurses in hospital and primary care largely occurs through informal means of communication [22–24]. Thus, it is reasonable to suggest that information exchange may be maintained by verbal communication, e.g. telephone reports. Nevertheless, we have not found any studies that investigated the instance of verbal information exchange between these settings.

Electronic exchange of information between hospital and primary care is under implementing in Norway, however, today most nursing summaries exchanged between hospital and home care are still paper-based [25] and most often transferred by the patient or by ordinary mail [22]. According to the guidelines [26, 27], the nursing summary should include descriptions of the nursing care delivered, the patient's status, as well as assessments and recommendations for continuing care. In addition to formal regulations and requirements, nursing education and practice are underpinned by values focusing on individual and holistic care of those who are ill [28–30]. In clinical practice, the holistic approach has been emphasized especially in palliative care [31]. However, it has been suggested that holistic care, or a palliative care approach, should form the basis of all care for older persons, also for those whose condition is not terminal [32]. This implies that nursing documentation, as well as nursing information exchange, should include a holistic approach, i.e. care that is related to the affective and functional needs of patients, where physical, psychological, social and spiritual/existential needs are all of importance. In contrast, studies have shown that nurses document physical caring needs more often than the other caring dimensions [13, 33–35]. In addition, it has been reported that nurses have being too focused on describing their tasks in the documentation rather than describing the patients’ needs and experiences [34, 36]. The findings of Jefferies et al. [37], which compared the content of nursing documentation and clinical handover, suggest that nurses’ verbal communication produces a more holistic view of the patient in which the patient's voice can be present. Their explanation was that nurses incorporate information from a wider variety of sources in the handover, which allows the nurse to present more contextual information in the communication. This may also apply to the information exchange between hospital and home care nurses. However, we failed to find any studies that investigated the content of verbal communication between these settings.

To our knowledge, there is a lack of studies investigating the entire communication loop during an episode of hospitalization of home care patients, i.e. both written and verbal communication, and from day of admission to return home (home care–to–hospital–to–home care). When investigating the whole process, we can picture the total information flow between the nurses, which could support nurses in improving an optimal and safe patient transfer. This would be an important element in the continuum of care and for the integration of services between primary and secondary care.

### Aim

The objective of the study was to identify and describe the process and content of the patient information exchange between nurses in home care and nurses at hospital during hospitalization of older home-living patients.

## Method

A multiple case study design was used. Yin [38:18] defines a case study as ‘an empirical inquiry that investigates a contemporary phenomenon in depth and within its real-life context, especially when the boundaries between phenomenon and context are not clearly evident’. When using a multiple case study design, the researcher is enabled to explore differences within and between cases and to replicate findings across the cases [38]. In this study, the home care and hospital nurses’ information exchange during each patient's episode of hospitalization, from day of admission (start of the case) to return home, was treated as a separate case.

### Setting and participants

The Norwegian health care system is divided into two organizational structures. While primary health care (including general practitioner, nursing homes, public health nurses and home care) is provided under the responsibility of local municipalities, the national health authorities owns and runs the hospitals organized within the specialist health care system. The organizational structure of home care is based on modified principles of new public management, including a purchaser–provider split [39]. This implies that while the purchaser unit assesses needs, formulates contracts, orders services and controls outcomes, the provider unit delivers care specified by the purchaser. The home care service includes both home nursing care and/or practical help. In the current study, home care is defined as home nursing care.

The study was conducted between September 2010 and July 2011 at two medical wards at a local hospital in central Norway and at a home care agency affiliated with this hospital. The reason for including these wards was that they were considered to be the wards with most inpatients from the home care.

Nine cases were included. Selection of cases was determined by the following inclusion criteria: patient 70+ years of age, consent competent, recipient of home care services and admitted from home and discharged back to home after the hospital treatment. Contact persons at the hospital ward recruited the patients and gained patient consent before the data collection started. Prior to gaining consent, they gave written and oral explanation of the study to the patients, including that focus was on nursing communication and that the patients would not be directly involved in the study. Finally, the patients who constituted the basis of the cases included one man and eight women with a median age of 85 years (range 70–95). Characteristics of the patients are shown in Table 1.

Prior to the data collection period, all the nurses in the home care agency and in the hospital wards received a letter of invitation to participate with an information sheet explaining the study. Informed consent was obtained from each nurse who participated. A total of 21 nurses, 1 man and 20 women, participated either directly through interviews and observations or indirectly as authors of the nursing documentation in the electronic patient record (EPR). Their median age was 40 (range 23–58 years). Their nursing experience varied from 0 to 35 years (median = 13 years). Five of the nurses specialized in geriatric nursing.

### Data collection

**Figure 1.**
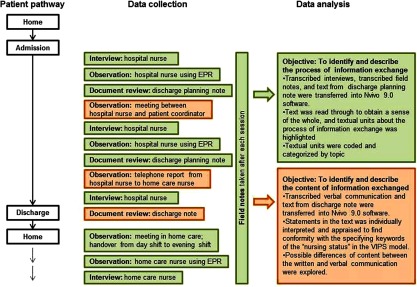
Overview of data collection and analysis, exemplified by Case 1.

Alternating between observations, qualitative interviews and document reviews, we tried to capture the total patient information exchange during each patient's episode of hospitalization, from day of admission to return home. These data from different sources allowed us to triangulate our findings [40]. All data collection was conducted by the first author. The process of data collection and analysis is shown in Figure 1.

#### Observations

Nonparticipant observations were conducted of nursing activities related to information exchange, including meetings, telephone reports and reading and writing nursing documentation in the electronic patient record. In addition to gaining insight into when and how information was exchanged, an important purpose of the observations was to capture any instances of verbal exchange between the nurses. At an early stage of the data collection period it became clear that verbal communication occurs by telephone calls between nurses in hospital and home care and through meetings between hospital nurses and patient coordinator from the municipal purchaser unit. In order to catch the entirety of these conversations, they were audio-recorded and transcribed verbatim. Since we were able to record only the hospital nurses’ voice and not the voice of the home care nurse during the telephone reports, we tried to get the overall picture of the conversation by interviewing the hospital nurse immediately after the telephone call was ended. Handwritten field notes were taken during the data collection process (15 hours) and transcribed shortly after each session.

#### Interviews

Unstructured, open-ended interviews were conducted with hospital nurses and home care nurses who were involved in the care of the patient. The purpose of the interviews was to catch when and how the information was exchanged. Interviews with the hospital nurses were conducted during the patient's admission. The home care nurses were interviewed when they had been on the first home visit after the patient returned home, i.e. in the afternoon the day of patient discharge. All interviews (*n* = 30) took place on site during work, lasted on average 10 minutes, were audio-recorded and later transcribed verbatim.

#### Document reviews

Two types of nursing documents from each patient's record at the hospital were reviewed by the researchers. The first document type, nursing transfer document, constituted the data for analyzing the written patient information exchange. The design of the transfer document comprised two main sections. The first section contained predefined information about the patient, relatives, providers and length of hospitalization. The second section, ‘patient's current status’, was open for the nurses to record free text within a structure in accordance with the research-based VIPS model. The VIPS model (an acronym formed from the Swedish words for well-being, integrity, prevention and security) provides a guiding framework for systematic nursing documentation with keywords on two levels, based on the concept of the nursing process [41, 42]. The model reflects and conceptualizes the essential elements of nursing practice and gives attention to individualized care. Only this second section of the transfer document, ‘patient's current status’, was included for investigation of the written patient information exchange. The second document type, the nursing discharge planning note, was used to capture when and how information was exchanged. The nursing discharge planning note is a tool for hospital nurses to plan the patients’ discharge from hospital and includes notes regarding contact and information exchange with expected providers after discharge.

### Data analysis

In order to identify the process of the information exchange, i.e. when and how the home care and hospital nurses exchange patient information, the transcribed interviews, discharge planning note and field notes were analyzed using qualitative content analysis [43].

To identify and describe the content of patient information exchanged, the written and verbal communications were scrutinized using both qualitative and quantitative content analysis [43]. First, text from the transfer documents (i.e. the section ‘patient's current status’, which was open for the nurses to record free text), and transcripts from the verbal communication were transferred verbatim into Nvivo 9.0 software. Then, using qualitative content analysis, every note/statement in the text was individually interpreted and appraised to find conformity with the specifying keywords of the ‘nursing status’ found in the VIPS model. These keywords are shown in Figure 3 in the Results section. Although VIPS initially was created for structuring written text, we consider it an appropriate framework for this study, as the verbal communication between nurses during patient transfer is seen as complementary to the written communication. After identifying statements in conformity with VIPS keywords, the analysis aimed to explore possible differences of content between the written and verbal communication. Using quantitative content analysis, the frequency of cases, which included statements in conformity with the VIPS keywords, was counted and compiled into a table.

Data collection and analysis were developed together in an iterative process whereby initial data collection informed future data collection and analysis within each case. After analyzing each case, a cross-case analysis was conducted to identify common themes among the nine cases. A review of the data as a whole, as well as the content analysis, was made by the third and last author to ensure trustworthiness [40].

### Ethical consideration

The Committee for Medical and Health Research Ethics of Norway approved the research project (no. 2009/815). Permission to perform data collection was obtained from the hospital research unit and the health administration in the municipality. All patients and nurses gave written informed consent after explanations of the study.

## Results

### The process of patient information exchange

**Figure 2.**
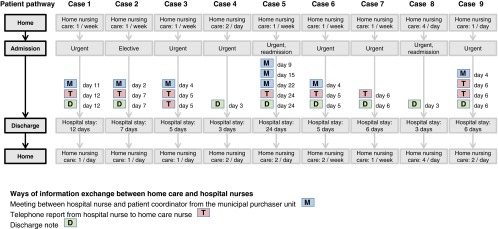
Overview of patient pathway and process of information exchange between home care nurses and hospital nurses.

Overview of the patient pathway and process of information exchange between home care nurses and hospital nurses is shown in Figure 2. The analysis of interviews, discharge planning notes and field notes revealed that information exchange mainly occurs at discharge from hospital to home care. In none of the cases was there information provided from the home care nurses to the hospital nurses.

In summary, six of the cases included both written and verbal information exchange, two only written, and one only verbal. Three ways of communication were identified. First, written in the form of a discharge note sent from hospital to home care. This was sent in paper version with the patients when they went back home, and later picked up by the home care nurse who first visited the patient. Nursing discharge notes were sent for 8 of the cases, ranging from 112 to 818 words (*M* = 382, Median = 343). Second, telephone reports were made by the hospital nurses to home care the same day as the patients were discharged. In most cases, the hospital nurses spent more time getting in touch with the right person, than time spent on the conversation. Telephone reports were made in seven of the cases, two of which were made twice, and lasted an average of 3 minutes (range 1½ to 10½ minutes). In all instances, the report was received in the home care by a day-shift nurse, who documented in the electronic patient record and handed over the information verbally to the evening-shift nurse who was taking the first visit after the patient returned home. A third way of communication was by meetings between hospital nurse and patient coordinator (a nurse) from the municipal purchaser unit. The purpose with these meetings is to provide information as both a basis for service allocation in the home care and preparing home care nurses to provide appropriate follow-up care. The meetings occur regularly twice a week. However, only those patients that the hospital nurses consider to have new or changing needs are brought up in the meetings, including six of the patients in this study, one of whom, with the longest hospital admission, was brought up at three occasions. The meetings lasted an average 6½ minutes per patient (range 2–11 minutes). The patient coordinator informed the home care after the meeting in two ways: verbally, either to the nursing manager, or to the day-shift nurse, and by case note documentation (in the electronic patient record). In none of the cases did the home care nurses read their patients case notes, although they had access to them.

While the discharge note constitutes a one-way communication, the telephone report and the meeting enable a two-way communication. However, the meeting must be regarded as indirect communication, where the municipal patient coordinator, who usually does not know the patient, acts as a mediator between the nurses in hospital and home care services.

### The content of information

**Figure 3.**
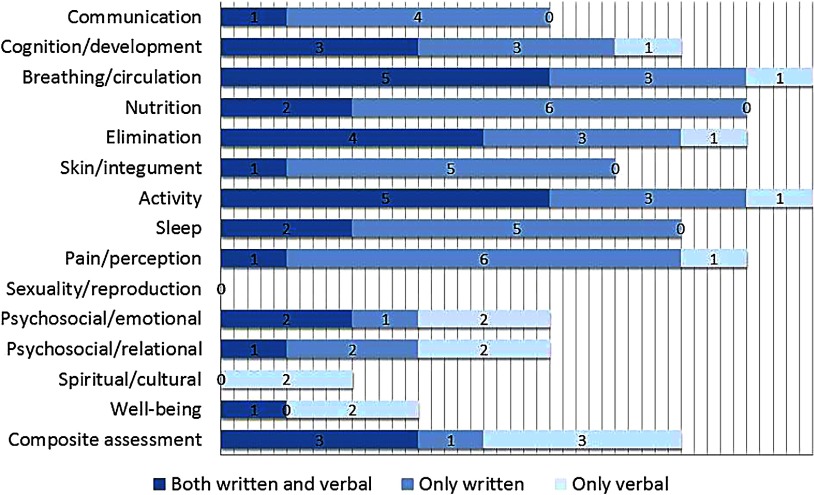
Cases with statements in conformity with VIPS keywords identified in written and verbal communication.

The number of cases, which included statements in conformity with the VIPS keywords, is shown in Figure 3. When considering the written and verbal communication together, information regarding ‘breathing/circulation’ and ‘activity’ were most frequently reported and were included in all nine cases. On the other hand, there was no information at all in any of the cases about ‘sexuality/reproduction’. When looking only at the written communication, information regarding ‘breathing/circulation’, ‘activity’ and ‘nutrition’ were most often exchanged, while ‘composite assessment’, ‘breathing/circulation’ and ‘activity’ were the most frequent topics of verbal communication. As shown in Figure 3, there was great variation in what was communicated in written or verbal form. What is especially noticeable is the keyword ‘spiritual/cultural’, which is the only topic that solely occurs in verbal communication. The statements concerned the patients’ experience of hopelessness and thoughts about the future, and included the two patients who had the longest hospital stay.

When looking at instances where VIPS keywords were communicated in both orally and writing, the content was to a large extent congruent. However, main differences of content were found for the keywords ‘composite assessment’ and ‘psychosocial’. For ‘composite assessment’, information given in verbal form was to a large extent more detailed than that given in the discharge note, especially when it comes to administration and prescription of medications. In the discharge notes, medication treatment was mainly described in short phrases, e.g. ‘*Antibiotic treatment for pneumonia*’. In verbal communication, more details were given, e.g. ‘*During the hospitalization, she has been treated with Antibiotics for her pneumonia, and has to continue the treatment for the next seven days, two tablets twice a day. I'll send the prescription with the patient so you can pick up the medicine at the pharmacy in the afternoon*’.

As regards the VIPS keyword ‘psychosocial’, the information concerning emotions was expressed shortly in an objective manner in the discharge notes, e.g. ‘*The patient is perceived as being in a good mood*’. Statements in the verbal communication, however, focused more on the patients’ subjective experience, and described both reason and consequences for feeling anxious and worrying, e.g. ‘*When she wakes up breathless from the chronic obstructive pulmonary disease at night, she says, she is feeling anxious and has to stand up from the bed and lie down on the sofa in the living-room. She is looking forward to coming back home, she says, but is worried about being alone in such situations*’. Differences were also found for statements regarding relations. In the discharge notes, the relations to relatives were mainly described quantitatively by short phrases, e.g. ‘*Has been visited several times by her son*’. The verbal information contained more details about the quality of the relations, e.g. ‘*He (the patient's husband) begins to be exhausted, because he spends so much time and effort caring for her at home*’.

## Discussion

To our knowledge, this is the first published study to explore both written and verbal patient information exchange between nurses in home care and hospital during hospitalization of older home-living patients. When following the entire communication loop for the nine cases of patient hospitalizations included in our sample, we found a gap in the information flow, as well as incompleteness in the content of information exchanged. From our findings, we argue that there is a lack of continuity of care during hospitalization of older home-living patients.

### The process of patient information exchange

Communication between the home care nurses and the hospital nurses mainly took place at discharge, and for none of the patients was information exchanged at admission. This means that we are not talking about a communication loop in terms of an uninterrupted information flow following the total patient pathway, i.e. from home care–to–hospital–to–home care. Instead, we are talking about information exchange with the starting point in the middle of the patient pathway, i.e. after the patient has been hospitalized, and which ends at the patient's discharge back to home care.

Written information exchange occurred only at discharge, including a nursing discharge note sent from hospital to home care at the moment the patient went home. This is in accordance with our previous study of prevalence of nursing transfer documents [20]. As nurses in primary and secondary care have reported in previous studies that they often use telephone [22–24], we were surprised by the fact that verbal information was not provided from the home care nurses in our sample. Unlike these previous studies, however, the design of the present study allowed us to follow the nurses’ information management within its real-life context and related to specific patient transfers. Indeed, the hospital nurses said they usually obtain collateral information from the home care by phone, but no phone calls were made at or just after admission of the included patients. Perhaps there may be a disparity between what the nurses say they do about information management and what they actually do, described by Argyris and Schön [44] in terms of theory-in-use and espoused theory of action. Nevertheless, although meetings and telephone conversations enabling two-way communication occurred towards the end of the hospitalizations, this could not have compensated for the lack of information at admission and prepared the hospital nurses for taking care of the patients. In addition, these conversations were led by the hospital nurses, and the focus was on discharge and not on taking care of the patient during hospitalization. In the study by Hellesø et al. [22], hospital nurses stated that they most often received patient details from the patients themselves and the patients’ relatives. However, we do not have information from the present study as to whether or not the hospital nurses received sufficient information from the patients themselves and/or their relatives.

The lack of information provided by home care nurses may have several explanations, for instance, lack of knowledge of the patient. Although the patients may have been home care recipients for perhaps months or years, nurses are not necessarily updated on the patients’ current status. Six of the patients included in our sample received assistance from home care only once a week prior to hospitalization. Often a high number of nursing staff are encountered in the care of the single patient [45], which may mean that months pass before a particular nurse sees the same patient. In such cases, available nursing documentation could have compensated for the lack of knowledge of the patient. However, documentation in home care has been reported as incomplete [46] and a major issue in acute situations [23, 47], which leads to another explanation; most of the patients in our sample were transferred urgently to the hospital. An urgent hospitalization is, in its nature, unexpected and hard to plan. In addition, the gathering and transfer of information may also be complicated due to a large physical distance between patients and their records [23], lack of time [47, 48], and nurses’ problems with reaching one another on the phone [49]. In some cases providers in primary care are even unaware that their patient is hospitalized [47, 50]. The patient may be admitted by an on-call physician who may not even know about the patient's affiliation to the home care. If the patient lives alone and is not accompanied by any relatives, which was the case for most of the patients in our sample, the issue of notifying the home care nurses largely depends on the patient's condition and ability to tell.

Information from the hospital to the home care was exchanged for all the patients in our sample, which was a pleasant finding considering the information needs of nurses to provide follow-up care after discharge. Most of the cases included both verbal and written information exchange at discharge, a finding in accordance with the study of Hellesø et al. [22], where nurses in home care and hospital assessed telephone and written information as the most-used communication tools. However, for one of the cases, information was given solely by verbal communication. Verbal information exchange may have some disadvantages compared to written. While the written information provides a wide platform for the storage of knowledge because the work of memory and conservation is inherent in the written word [51], the verbal information is reliant on memory and the details of the information may be omitted or forgotten. The use of meetings between hospital nurse and municipal patient coordinator as a tool for exchanging information may have some disadvantages. As the patient coordinator acts as a mediator between the nurses, there is a risk that the information may be distorted before it reaches the home care nurses. In addition, as the patient coordinator usually does not know the patient, facility follow-up questions that are crucial for the continuity of care might be omitted. Furthermore, in the role as purchaser in the municipality, the patient coordinator is to be the driving force for increased productivity and efficiency in the home care [52]. In order to keep down the allocation of services, perhaps there is a underestimating of the patient's needs when passing the information to the home care nurses. In a study by Dale and Hvalvik [53], home nursing leaders expressed frustration and worries about the responsibility for deciding and planning home care was placed on the purchaser unit. They experienced that the patients often returned home without home care being involved, which left them uninformed and unprepared. Anyway, it may be problematic if the hospital nurse relies on the patient coordinator's ability to forward information to the home care, and thereby does not make effort on providing a comprehensive nursing discharge note. The home care nurses in our sample did not read the case note documentation made by the patient coordinator from the purchaser unit. As they did not have access to the nursing discharge note before they picked it up at the patient's home, they had to rely solely on the information provided verbally.

### The content of information exchange

Considering the written and verbal communication together, and the written communication isolated, information regarding physical care was more frequently reported than other caring dimensions in our sample. This is a finding in accordance with previous studies that have investigated nurses’ written information [13, 34, 35]. When looking only at the verbal communication, the amount of statements in conformity with the VIPS keyword ‘composite assessment’ was just as high as for the physically related keywords ‘breathing/circulation’ and ‘activity’. This could be explained by hospital nurses’ belief in this information, e.g. administration and prescription of medicaments and technical aids have to be in place before the patient arrives. As the discharge note is printed out and sent with the patient when he/she returns home, its content is not available for the home care nurses to make preparations in advance. This could explain why the verbal communication concerning ‘composite assessment’ was more detailed, and included messages such as ‘*pick up the medicine at the pharmacy*’.

In line with other studies [13, 33, 35], we found a lack of information regarding patients’ ‘spiritual/cultural’ needs in the nursing documentation. However, we found that this topic was maintained in some of the verbal communication. As this included the patients who had the longest hospital stay, this finding may suggest that nurses have to be familiar with the patient before they can observe and report on this topic. An explanation for why this information was not included in the discharge note may be that spiritual and existential needs are considered as a private matter and closely connected to the subjective experience. In our study, descriptions of the patients’ subjective experiences were almost absent and occurred only in the verbal communication. According to Kärkkäinen et al. [36], the lack of focus on the patient's experiences in the nursing documentation may be due to the nurses’ propensity to describe the patient's problems and needs without conferring with the patient. The findings of Jefferies et al. [37] suggest that nurses have a more holistic view of the patient in verbal communication because they incorporate information from a wider variety of sources which allows the nurse to present more contextual information in the communication.

The major focus on physical care at the expense of other caring dimensions in our sample may be explained by biomedical overlay in the context of hospital care – the ‘voice of medicine’ [34]. However, this may also reflect a lack of knowledge and skills in responding to other caring needs in actual nursing practice. Nurses have reported that they consider their capacity to meet the psychosocial needs of older people as less adequate compared with the physical needs [54].

Haggerty and colleagues’ framework states that informational continuity occurs when all relevant patient events and circumstances are known to providers [16, 17]. Yet, for none of the patients included in our study was the patient's health information able to ‘follow him/her’ throughout the entire pathway of care. The breakdown in communication between nurses, particularly at patient admission, raises a concern about the continuity of care to older patients transferred between home care and hospital. As change in condition can be a sign of acute disease [55–57], and physical and mental failures necessitate specific approaches to clinical care [58, 59], exchanging information about the older patient's holistic health status and usual condition is significant to ensure appropriate care at the next health care setting. Previous studies in this area have to a large extent focused on information exchange during the discharge from hospital. Based on our findings, we recommend that further research devote more attention to the nurses’ communication at admission.

### Strengths and limitations of the study

A case study design is well suited when the purpose is to describe the holistic and meaningful aspects of real-life events and contexts [38]. In our study, several data sources were collected to deepen our understanding of nurses’ patient information exchange. As we used a multiple case study design, we were enabled to explore differences within and between cases and to replicate findings across the cases.

The fact that the transfer documents were retrieved from the patient records at the hospital may have constituted a limitation, as we only had the opportunity to identify transfer documents that were received by and sent from the hospital. However, by interviewing the home care nurses we could verify that no documents had been generated and then lost on the way at admission. Another limitation concerns the fact that we were able to record only the hospital nurses’ voice and not the voice of the home care nurse during the telephone reports. However, we tried to get the overall picture of the conversations by interviewing the hospital nurse immediately after the telephone call was ended. Due to the small sample size, our findings are not necessarily generalizable to the broader population, but they inform our theoretical propositions of nurses’ information exchange [38]. As we triangulated the data sources, and provided rich descriptions of the information exchange related to each patient, validity of the results increased [38]. Finally, a limitation may be that participating nurses may have demonstrated an increased awareness in their information exchange as a result of being observed.

## Conclusion

The gap in the information flow, as well as incompleteness in the content of written and verbal information exchanged, constitutes a challenge to the continuity of care for hospitalized home-living patients. In order to ensure appropriate nursing follow-up care, we emphasize the need for nurses to improve the information flow, as well as to use a more comprehensive approach to older patients, and that this must be reflected in the verbal and written information exchange.

## Figures and Tables

**Table 1.**
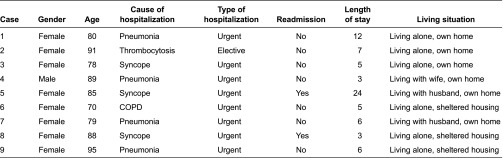
Overview of included patients

## References

[1] Marengoni A, Angleman S, Melis R, Mangialasche F, Karp A, Garmen A (2011). Aging with multimorbidity: a systematic review of the literature. Ageing Research Reviews.

[2] Nardi R, Scanelli G, Corrao S, Iori I, Mathieu G, Cataldi Amatrian R (2007). Co-morbidity does not reflect complexity in internal medicine patients. European Journal of Internal Medicine.

[3] Condelius A, Edberg AK, Jakobsson U, Hallberg IR (2008). Hospital admissions among people 65+ related to multimorbidity, municipal and outpatient care. Archives of Gerontology and Geriatrics.

[4] Vogeli C, Shields AE, Lee TA, Gibson TB, Marder WD, Weiss KB (2007). Multiple chronic conditions: prevalence, health consequences, and implications for quality, care management, and costs. Journal of General Internal Medicine.

[5] Reed J, Cook G, Childs S, McCormack B (2005). A literature review to explore integrated care for older people. International Journal of Integrated Care [serial online].

[6] Coleman EA (2003). Falling through the cracks: challenges and opportunities for improving transitional care for persons with continuous complex care needs. Journal of the American Geriatrics Society.

[7] Norwegian Ministry of Health and Care Services Stortingsmelding nr. 47 (2008–2009), Samhandlingsreformen. Rett behandling – på rett sted – til rett tid.

[8] Garåsen H, Johnsen R (2007). The quality of communication about older patients between hospital physicians and general practitioners: a panel study assessment. BMC Health Services Research [serial online].

[9] Boockvar KS, Fridman B, Marturano C (2005). Ineffective communication of mental status information during care transfer of older adults. Journal of General Internal Medicine.

[10] Cwinn MA, Forster AJ, Cwinn AA, Hebert G, Calder L, Stiell IG (2009). Prevalence of information gaps for seniors transferred from nursing homes to the emergency department. Canadian Journal of Emergency Medicine.

[11] Carlsson E, Ehnfors M, Eldh AC, Ehrenberg A (2012). Accuracy and continuity in discharge information for patients with eating difficulties after stroke. Journal of Clinical Nursing.

[12] Glintborg B, Andersen SE, Dalhoff K (2007). Insufficient communication about medication use at the interface between hospital and primary care. Quality & Safety in Health Care.

[13] Hellesø R, Lorensen M, Sorensen L (2004). Challenging the information gap – the patients transfer from hospital to home health care. International Journal of Medical Informatics.

[14] Mesteig M, Helbostad JL, Sletvold O, Rosstad T, Saltvedt I (2010). Unwanted incidents during transition of geriatric patients from hospital to home: a prospective observational study. BMC Health Services Research [serial online].

[15] Heaton J, Corden A, Parker G (2012). ‘Continuity of care’: a critical interpretive synthesis of how the concept was elaborated by a national research programme. International Journal of Integrated Care [serial online].

[16] Haggerty JL, Reid RJ, Freeman GK, Starfield BH, Adair CE, McKendry R (2003). Continuity of care: a multidisciplinary review. British Medical Journal.

[17] Freeman GK, Woloshynowych M, Baker R, Boulton M, Guthrie B, Car J (2007). Continuity of care 2006: what have we learned since 2000 and what are policy imperatives now?. Report for the National Co-ordinating Centre for NHS Service Delivery and Organisation R&D.

[18] Norwegian Ministry of Health and Care Services Lov om helsepersonell av 2.juli 1999 nr 64. Helsepersonelloven.

[19] Norwegian Ministry of Health and Care Services (2000). Forskrift om pasientjournal.

[20] Olsen RM, Hellzèn O, Enmarker I (2013). Nurses' information exchange during older patient transfer: prevalence and associations with patient and transfer characteristics. International Journal of Integrated Care [serial online].

[21] Björvell C, Wredling R, Thorell-Ekstrand I (2002). Long-term increase in quality of nursing documentation: effects of a comprehensive intervention. Scandinavian Journal of Caring Science.

[22] Hellesø R, Sorensen L, Lorensen M (2005). Nurses' information management across complex health care organizations. International Journal of Medical Informatics.

[23] Kihlgren AL, Fagerberg I, Skovdahl K, Kihlgren M (2003). Referrals from home care to emergency hospital care: basis for decisions. Journal of Clinical Nursing.

[24] Hellesø R, Rostad HM, Gilstad H, Melby L, Jaatun MG Interaction climate and use of information systems in discharge planning: Hospital nurses’ perspectives. Proceedings of the European workshop on practical aspects of health informatics (PAHI 2013), Edinburgh, Scotland, UK, 2013 Mar 11.

[25] Norwegian Ministry of Health and Care Services (2008). Samspill 2.0: nasjonal strategi for elektronisk samhandling i helse- og sosialsektoren 2008–2013.

[26] KITH (2003). Kravspesifikasjon for elektronisk dokumentasjon av sykepleie. Nasjonal standard.

[27] The Norwegian Nursing Organisation (2007). Dokumentasjon av sykepleie i elektronisk pasientjournal.

[28] Povlsen L, Borup IK (2011). Holism in nursing and health promotion: distinct or related perspectives? – A literature review. Scandinavian Journal of Caring Science.

[29] McEvoy L, Duffy A (2008). Holistic practice – a concept analysis. Nurse Education in Practice.

[30] International Council of Nurses (2012). The ICN Code of Ethics for Nurses (revised 2012).

[31] Davies E, Higginson IJ (2004). Better palliative care for older people.

[32] Hallberg IR (2006). Palliative care as a framework for older people's long-term care. International Journal of Palliative Nursing.

[33] Olsen RM, Hellzèn O, Skotnes LH, Enmarker I (2012). Content of nursing discharge notes: Associations with patient and transfer characteristics. Open Journal of Nursing.

[34] Hyde A, Treacy MP, Scott PA, Butler M, Drennan J, Irving K (2005). Modes of rationality in nursing documentation: biology, biography and the ‘voice of nursing’. Nursing Inquiry.

[35] Gunhardsson I, Svensson A, Bertero C (2008). Documentation in palliative care: nursing documentation in a palliative care unit – a pilot study. The American Journal of Hospice & Palliative Care.

[36] Kärkkäinen O, Bondas T, Eriksson K (2005). Documentation of individualized patient care: a qualitative metasynthesis. Nursing Ethics.

[37] Jefferies D, Johnson M, Nicholls D (2012). Comparing written and oral approaches to clinical reporting in nursing. Contemporary Nurse.

[38] Yin RK (2003). Case study research: design and methods. Applied social research methods series.

[39] Vabø M (2012). Norwegian home care in transition – heading for accountability, off-loading responsibilities. Health and Social Care in the Community.

[40] Patton MQ (2002). Qualitative evaluation and research methods.

[41] Ehnfors M, Thorell-Ekstrand I, Ehrenberg A (1991). Towards basic nursing information in patient records. Vård i Norden.

[42] Ehrenberg A, Ehnfors M, Thorell-Ekstrand I (1996). Nursing documentation in patient records: experience of the use of the VIPS model. Journal of Advanced Nursing.

[43] Krippendorff K (2004). Content analysis: an introduction to its methodology.

[44] Argyris C, Schön DA (1974). Theory in practice: increasing professional effectiveness.

[45] Gjevjon ER, Eika KH, Romøren TI, Landmark BF (2014). Measuring interpersonal continuity in high-frequency home healthcare services. Journal of Advanced Nursing.

[46] Gjevjon ER, Helleso R (2010). The quality of home care nurses' documentation in new electronic patient records. Journal of Clinical Nursing.

[47] Olsen RM, Østnor BJ, Enmarker I, Hellzén O (2013). Barriers to information exchange during older patients' transfer: nurses' experiences. Journal of Clinical Nursing.

[48] Meissner A, Hasselhorn HM, Estryn-Behar M, Nezet O, Pokorski J, Gould D (2007). Nurses' perception of shift handovers in Europe: results from the European Nurses' Early Exit Study. Journal of Advanced Nursing.

[49] Olsson M, Larsson LG, Flensner G, Back-Pettersson S (2012). The impact of concordant communication in outpatient care planning – nurses' perspective. Journal of Nursing Management.

[50] Bell CM, Schnipper JL, Auerbach AD, Kaboli PJ, Wetterneck TB, Gonzales DV (2009). Association of communication between hospital-based physicians and primary care providers with patient outcomes. Journal of General Internal Medicine.

[51] Ong WJ (2012). Orality and literacy: the technologizing of the word.

[52] Vabø M (2009). Home care in transition: the complex dynamic of competing drivers of change in Norway. Journal of Health Organization and Management.

[53] Dale B, Hvalvik S (2013). Administration of care to older patients in transition from hospital to home care services: home nursing leaders' experiences. Journal of Multidisciplinary Healthcare [serial online].

[54] Isola A, Backman K, Voutilainen P, Rautsiala T (2008). Quality of institutional care of older people as evaluated by nursing staff. Journal of Clinical Nursing.

[55] Gray LK, Smyth KA, Palmer RM, Zhu X, Callahan JM (2002). Heterogeneity in older people: examining physiologic failure, age, and comorbidity. Journal of the American Geriatrics Society.

[56] Chong CP, Street PR (2008). Pneumonia in the elderly: a review of the epidemiology, pathogenesis, microbiology, and clinical features. Southern Medical Journal.

[57] Cerejeira J, Mukaetova-Ladinska EB (2011). A clinical update on delirium: from early recognition to effective management. Nursing Research and Practice [serial online].

[58] Ornstein K, Smith KL, Foer DH, Lopez-Cantor MT, Soriano T (2011). To the hospital and back home again: a nurse practitioner-based transitional care program for hospitalized homebound people. Journal of the American Geriatrics Society.

[59] Boling PA (2009). Care transitions and home health care. Clinics in Geriatric Medicine.

